# Genomic and functional characterization of ST11-KL64 hypervirulence-associated carbapenem-resistant *Klebsiella pneumoniae* co-harboring *bla*_KPC-2_ and *bla*_NDM-13_

**DOI:** 10.3389/fmicb.2026.1874278

**Published:** 2026-08-25

**Authors:** Haojun Chen, Liping Liu, Lijun Huang, Jie Huang, Tingting Li, Xiaoxue Huang, Qiurong He

**Affiliations:** Department of Laboratory Medicine, West China School of Public Health and West China Fourth Hospital, Sichuan University, Chengdu, China

**Keywords:** *bla*_KPC-2_, *bla*_NDM-13_, hypervirulence-associated carbapenem-resistant *Klebsiella pneumoniae*, IncI1 plasmid, ST11-KL64

## Abstract

**Background:**

Hypervirulence-associated carbapenem-resistant *Klebsiella pneumoniae* (hv-CRKP) is a major clinical and public health threat. However, ST11-KL64 hv-CRKP co-harboring *bla*_KPC-2_ and *bla*_NDM-13_ remains poorly characterized, particularly regarding genomic relatedness, plasmid dynamics, and attenuated virulence-associated phenotypes.

**Methods:**

We retrospectively investigated clinical *K. pneumoniae* isolates collected at a tertiary hospital in Chengdu, China, between January and December 2024. Hypervirulence-associated markers were screened by PCR, followed by antimicrobial susceptibility testing and carbapenemase inhibitor enhancement assay to identify genotype-defined hv-CRKP. All isolates were subjected to molecular typing. ST11-KL64 isolates co-harboring *bla*_KPC-2_ and *bla*_NDM-13_ were subjected to Illumina sequencing, with the representative isolate K3 undergoing hybrid whole-genome sequencing and functional characterization.

**Results:**

Among the 46 hvKP isolates recovered from 43 patients, 35 were identified as hv-CRKP, predominantly ST11-KL64. Three ST11-KL64 hv-CRKP isolates co-harbored *bla*_KPC-2_/*bla*_NDM-13_, and Illumina sequencing coupled with core-genome SNP (cgSNP) typing revealed minimal genetic variation. The expanded cgSNP analysis supported close relatedness between K3 and Beijing isolate K56649. K3 carried a pLVPK-like virulence plasmid, a *bla*_KPC-2_-bearing IncFII/IncR plasmid, and a *bla*_NDM-13_-bearing IncI1 plasmid. Relative to pK2044, K3 exhibited an *rmpA*-proximal *ISKpn26*-associated insertion and a complex alteration of the 5′-terminal coding region of *rmpA*. The *bla*_NDM-13_ plasmid was conjugatively transferred to *Escherichia coli* C600 with a mean conjugation frequency of 5.213 × 10^−3^ transconjugants per recipient cell and *bla*_NDM-13_ maintained high stability following approximately 100 generations of antibiotic-free passage, whereas *bla*_KPC-2_ was not detected under the tested conditions. Phenotypically, K3 showed a negative string test, low mucoviscosity, and attenuated virulence-associated phenotypes.

**Conclusion:**

Our results reveal that the three isolates formed a closely related local genomic cluster, among which K3 was closely related to the K56649 clone. In addition, K3 exhibited conjugative transfer capacity of the *bla*_NDM-13_-bearing IncI1 plasmid, and alterations at the *rmpA* locus accompanied by reduced *rmpA* transcript abundance were associated with low mucoviscosity.

## Introduction

*Klebsiella pneumoniae* (KP) comprises two major pathotypes, classical *K. pneumoniae* (cKP) and hypervirulent *K. pneumoniae* (hvKP), with distinct clinical and biological features. Under antimicrobial selection pressure, cKP has frequently acquired carbapenemase-encoding plasmids and evolved into carbapenem-resistant *K. pneumoniae* (CRKP; Han et al., 2025; Luo et al., 2024; Lin et al., 2024). In contrast, hvKP is classically associated with severe invasive infections, even in otherwise healthy individuals (You et al., 2024; Piazza et al., 2022; Mohamed et al., 2025). The convergence of carbapenem resistance and hypervirulence has led to the emergence of hypervirulence-associated carbapenem-resistant *K. pneumoniae* (hv-CRKP), which poses a major public health threat (Heng et al., 2025; Pu et al., 2023; Liao et al., 2024).

Carbapenemase production is the main resistance mechanism in hv-CRKP, with *Klebsiella pneumoniae* carbapenemase (KPC), particularly KPC-2, being predominant (Wang et al., 2025; Cox et al., 2025). Although hv-CRKP isolates co-harboring *bla*_KPC-2_ and metallo-*β*-lactamase genes such as *bla*_NDM-1_ or *bla*_NDM-5_ have been reported (Huang et al., 2023; Zhou et al., 2024), *bla*_NDM-13_ remains much less frequently documented. NDM-13 was first identified in 2015 in chromosomally encoded form within a clinical ST101 *Escherichia coli* isolate recovered from Nepal (Shrestha et al., 2015). Relative to NDM-1, NDM-13 differs by two amino acid substitutions, D95N and M154L, and exhibits a broadly comparable *β*-lactam hydrolysis profile, although increased catalytic efficiency against cefotaxime has been reported. Subsequent studies identified plasmid-borne *bla*_NDM-13_ in different *Enterobacterales* hosts, including a self-transmissible IncX3 plasmid in *E. coli* and an IncI1 plasmid in *Salmonella* (Lv et al., 2016; Huang et al., 2022). Recently, (Zhang et al. 2024) described an outbreak of ST11-KL64 hv-CRKP co-harboring *bla*_KPC-2_ and *bla*_NDM-13_ in Beijing, while (Tao et al. 2025) reported an additional clinical isolate with the same genotype. These observations indicate that, despite its relatively low prevalence, *bla*_NDM-13_ has entered transferable plasmid platforms and high-risk epidemic ST11 *K. pneumoniae* lineage, raising concerns regarding local clonal expansion, plasmid-mediated dissemination, and further restriction of therapeutic options.

However, the genomic relatedness of ST11-KL64 isolates co-harboring *bla*_KPC-2_ and *bla*_NDM-13_, the genetic features and transferability of their *bla*_NDM-13_-bearing plasmids, and the extent to which their virulence-associated phenotypes remain incompletely understood. Here, we investigated three ST11-KL64 hv-CRKP isolates co-harboring *bla*_KPC-2_ and *bla*_NDM-13_ to characterize their local genomic relatedness and assess the relationship between representative isolate K3 and reported Chinese isolates. We further characterized the plasmid characteristics, assessed the transferability and stability of its *bla*_NDM-13_-bearing IncI1 plasmid, and examined the association between alterations at the *rmpA* locus and low mucoviscosity in K3.

## Materials and methods

### Clinical isolates and definitions

This retrospective study was conducted at the Fourth Hospital of West China, Sichuan University, a tertiary-care hospital in Chengdu, China. All *K. pneumoniae* isolates were recovered from routine clinical specimens submitted for microbiological culture between January and December 2024. Species identification was performed by matrix-assisted laser desorption/ionization time-of-flight mass spectrometry (MALDI-TOF MS; bioMérieux, France). Redundant isolates were defined as isolates recovered from the same patient and the same specimen type during the study period. Isolates were retained only if they were obtained from distinct clinically relevant infection sites; otherwise, only the first clinically relevant isolate was included. Colonization isolates were excluded after reviewing clinical records, especially clinical manifestations, available laboratory and imaging findings as well as clinical diagnosis and treatment regimens. Hypervirulence-associated marker screening was performed for *rmpA*, *rmpA2*, *iroB*, *iucA*, and *peg-344*. For cohort screening, isolates harboring at least one of these markers were classified as genotype-defined hvKP. Carbapenem-resistant isolates within this genotype-defined hvKP group were operationally designated as genotype-defined hv-CRKP.

### Screening of virulence, resistance, and molecular characteristics

DNA extraction, string test, detection of virulence-associated genes, capsular serotype determination based on *wzi* sequencing, antimicrobial susceptibility testing, carbapenemase phenotypic testing, carbapenemase gene detection, and multilocus sequence typing (MLST) were performed as previously described (Chen et al., 2025). The string test was performed as a phenotypic assay for hypermucoviscosity, and the results were recorded descriptively. It was not incorporated into the operational definition of genotype-defined hvKP or hv-CRKP. For the three ST11-KL64 genotype-defined hv-CRKP isolates co-harboring *bla*_KPC-2_ and *bla*_NDM-13_ and the transconjugant K3T, MIC profiles were confirmed again. MICs of tigecycline and polymyxin B were confirmed by broth microdilution, while automated system was used for the remaining antibiotics (except for aztreonam-avibactam). Antimicrobial susceptibility results were interpreted according to CLSI M100-ED36, except for tigecycline (FDA criteria). Amplification primers are listed in Supplementary Table S1.

### Whole genome sequencing and bioinformatics analysis

All three ST11-KL64 genotype-defined hv-CRKP isolates co-harboring *bla*_KPC-2_ and *bla*_NDM-13_ were sequenced using the Illumina platform, whereas K3 was additionally subjected to Nanopore sequencing for complete hybrid genome assembly. *De novo* assembly was performed using SPAdes v4.1.0 (Bankevich et al., 2012) and Unicycler v0.5.0 (Wick et al., 2017), and genome annotation was carried out using Prokaryotic Genome Annotation Pipeline (Tatusova et al., 2016). ST and KL were determined using Kleborate v3.1.3 (Lam et al., 2021). Plasmid replicon types were identified using PlasmidFinder v2.1.^1^ Virulence and antimicrobial resistance genes were predicted using the Virulence Factor Database (VFDB)^2^ and ResFinder v4.5.0,^3^ respectively. Predicted transfer-associated components of pK3-3-NDM were identified by oriTfinder (Li et al., 2018).

Comparative analysis of virulence and resistance plasmids was performed using BLAST Ring Image Generator (BRIG) v0.95 (Alikhan et al., 2011). Genetic environments surrounding *bla*_KPC-2_ and *bla*_NDM-13_ were compared using Easyfig v2.2.5 (Sullivan et al., 2011).

### cgSNP-based phylogenetic analysis

cgSNP analysis was performed using the chromosome of K3 (CP196480.1) as the reference genome. SNPs were called using Snippy v4.6.0,^4^ and the resulting core-genome alignment was subjected to recombination detection and filtering using Gubbins v3.3.1 (Croucher et al., 2015) with default parameters. SNPs located within Gubbins-predicted recombinant regions were excluded from downstream distance calculation and phylogenetic reconstruction. Pairwise SNP distances were calculated from the recombination-filtered core-genome alignment using SNP-dists v0.8.2,^5^ and phylogenetic reconstruction was performed using IQ-TREE v3.0.1 (Wong et al., 2026).

For local relatedness analysis, Illumina assemblies from K5 and K60 were mapped to the complete K3 chromosome, followed by SNP calling, recombination filtering, and pairwise cgSNP-distance calculation using the same pipeline described above. Publicly available *K. pneumoniae* genome assemblies were retrieved from the National Center for Biotechnology Information to provide three levels of genomic context. The core comparator group included all available ST11-KL64 genotype-defined hv-CRKP genomes co-harboring *bla*_KPC-2_ and *bla*_NDM-13_. The local background group included 16 available Sichuan genomes representing locally circulating genotype-defined hv-CRKP or CRKP. Contextual outgroups included 61 domestic and international genomes, comprising 43 ST11-KL64, 5 ST11-KL47, and 13 non-ST11-KL64/KL47 genomes. Genome quality was assessed using QUAST v4.4 and CheckM v1.0.18, as implemented within the KBase platform (Arkin et al., 2018). Assemblies were excluded if they had >100 contigs, an N50 < 100 kb, a maximum contig length <500 kb, genome completeness <96%, or contamination >2.0%. The final dataset included 80 genomes, and detailed metadata, group assignment, and assembly-quality metrics are provided in Supplementary Table S2.

### RT-qPCR

RT-qPCR was performed to quantify *rmpA* transcription in K3. Total RNA extraction, reverse transcription, and qPCR amplification were carried out strictly in accordance with the manufacturer’s instructions (TaKaRa, Dalian, China). qPCR primers were all located downstream of the structural alteration region and listed in Supplementary Table S3. Expression of *rmpA* was normalized to *rpoB* and samples with undetectable transcripts or values near the limit of detection were analyzed using ΔCt values rather than relative fold change. *K. pneumoniae* ATCC 700603 and hypervirulent *K. pneumoniae* NTUH-K2044 were included as controls. All experiments were performed with three biological replicates and three technical replicates.

### Conjugation and plasmid stability assay

Conjugation experiments were performed using K3 as the donor strain and rifampicin-resistant *E. coli* C600 as the recipient. Log-phase donor and recipient cells were mixed at a 1:3 ratio and incubated in LB broth at 37 °C for 6 h. Transconjugants were selected on China Blue agar containing meropenem (2 μg/mL) and rifampicin (100 μg/mL) and were confirmed by antimicrobial susceptibility testing, PCR, and Illumina sequencing. For determination of the meropenem MICs of K3 and K3T, an extended concentration range up to 512 μg/mL was used. Conjugation frequency was calculated as the number of transconjugants per recipient cell.

Plasmid stability was assessed by serial passaging without antibiotic selection (Gao et al., 2020). Briefly, a single colony was cultured overnight and passaged daily at 1:1,000 dilution for 10 consecutive days (approximately 100 generations). Thirty colonies were randomly selected from day 0 and day 10, respectively, were screened by PCR for *bla*_KPC-2_ and *bla*_NDM-13_ to determine plasmid retention.

### Virulence-associated phenotypes

Virulence-associated phenotypes were evaluated by mucoviscosity assay, siderophore production, serum resistance, and *Galleria mellonella* infection model, as previously described with minor modifications (Zhou et al., 2024; Zhang et al., 2024; Mike et al., 2021; Wang et al., 2024). For the mucoviscosity assay, single colonies were cultured in LB broth at 37 °C with shaking at 150 rpm for 18 h, and overnight cultures were normalized to an OD600 of approximately 1.0 with sterile phosphate-buffered saline. 5 mL of the normalized suspension was centrifuged at 1,000 × g for 5 min at room temperature, and the OD600 of the upper supernatant was measured. The mucoviscosity index was calculated as OD600-supernatant/OD600-total of the normalized suspension. Siderophore production was assessed on chrome azurol S (CAS) agar by spotting 1 μL of log-phase bacterial suspension (10^5^ CFU/mL) and measuring the diameter of the orange halo after incubation at 37 °C for 48 h. Serum resistance was determined by incubating bacterial suspension (10^6^ CFU/mL) with pooled normal human serum at 37 °C for 0 h, 1 h, 2 h, and 3 h, followed by colony counting at the indicated time points and grading into six levels as previously described (Mei et al., 2017). Grades 5–6, 3–4, and 1–2 were classified as resistant, intermediate, and sensitive, respectively. *In vivo* virulence was assessed using the *G. mellonella* model by inoculating larvae (approximately 250 mg) with 10 μL of log-phase bacterial suspension (10^7^ CFU/mL), followed by incubation at 37 °C and survival monitoring every 12 h for 72 h. Each group contained 10 larvae. ATCC 700603 and NTUH-K2044 were used as low- and high-virulence controls, respectively, and phosphate-buffered saline served as the blank control in the *G. mellonella* assay. All experiments were performed in biological triplicate.

### Statistical analysis

Statistical analyses were performed using R version 4.4.0. Clinical continuous variables were expressed as medians with interquartile ranges, and categorical variables as frequencies and percentages. The Mann–Whitney U test was used to compare continuous variables between groups, while categorical variables were compared using the chi-square test or Fisher’s exact test, as appropriate. Quantitative experimental data were presented as means and SD from three independent biological replicates and analyzed using one-way ANOVA followed by Tukey’s test. A two-sided *p* value < 0.05 was considered statistically significant.

## Results

### Identification of *bla*_KPC-2_ and *bla*_NDM-13_ co-carrying ST11-KL64 genotype-defined hv-CRKP isolates

**Figure 1:**
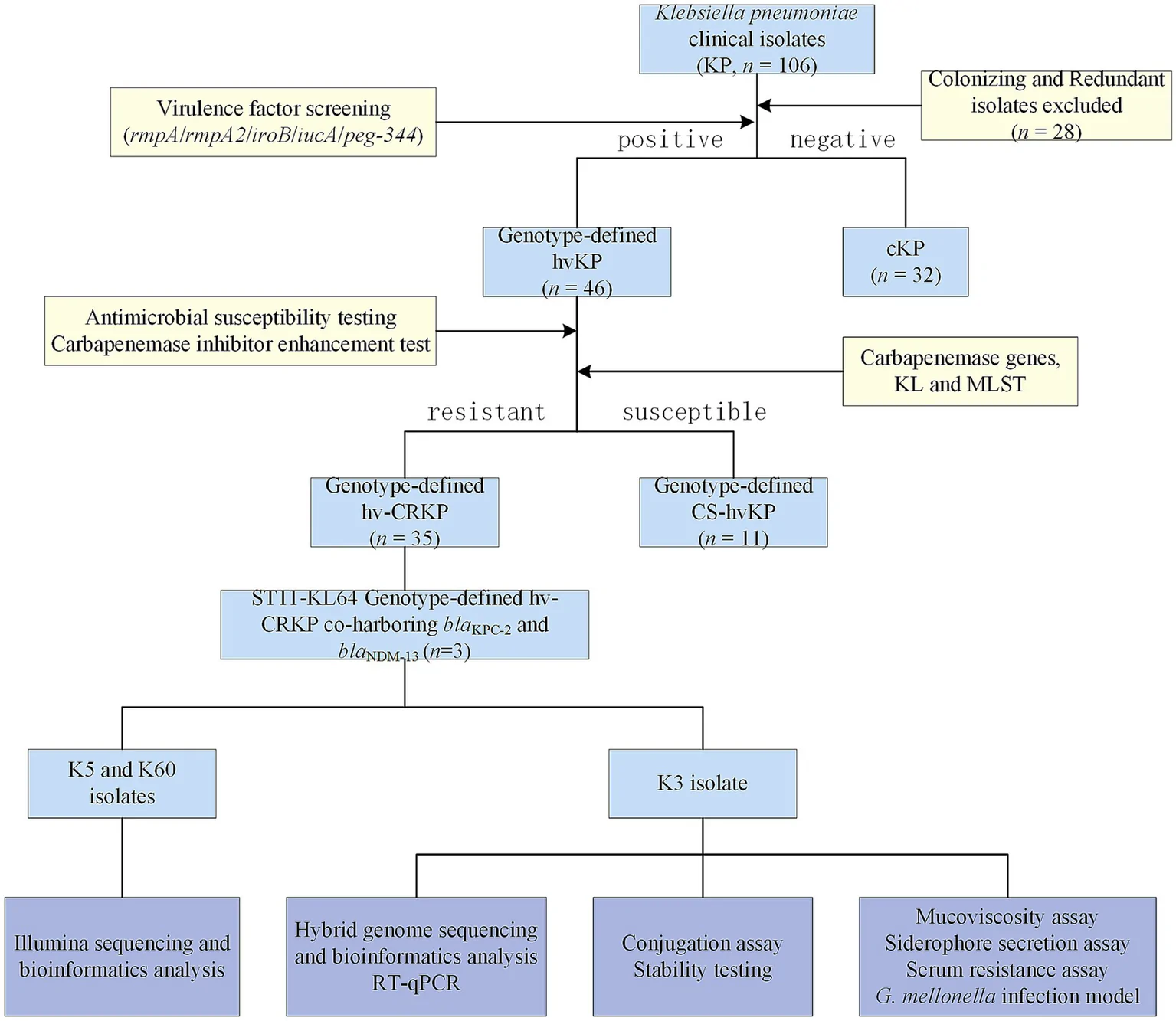
Flowchart of inclusion and exclusion criteria for 106 *K. pneumoniae* strains.

As shown in Figure 1, among an initial collection of 106 *K. pneumoniae* isolates, 46 genotype-defined hvKP isolates from 43 patients were identified after exclusion of colonizing isolates and duplicates. Clinical characteristics are summarized in Table 1. ICU admission rates were comparable between patients with genotype-defined hv-CRKP and carbapenem-susceptible hypervirulent *K. pneumoniae* (CS-hvKP). However, invasive procedures were more common in the genotype-defined hv-CRKP group, including tracheostomy (62.5% vs. 27.3%, *p* = 0.043) and indwelling gastric tube placement (53.1% vs. 9.1%, *p* = 0.014). 30-day all-cause mortality did not differ significantly between groups, although mortality in the genotype-defined hv-CRKP group reached 34.4%.

**Table 1:** Clinical characteristics of 43 patients with genotype-defined hvKP infection.

Characteristic	hv-CRKP (*n*, %) (*n* = 32)	CS-hvKP (*n*, %) (*n* = 11)	*p*-value
Basic demographics			
Age^a^	62 (45, 73)	52 (32, 59)	0.191
Male	19 (59.4)	6 (54.5)	1.000
Departments			
Admitted to ICU	18 (56.3)	4 (36.4)	0.255
Underlying diseases			
Diabetes	8 (25.0)	3 (27.3)	1.000
Hypertension	16 (50.0)	3 (27.3)	0.294
Cardiovascular disease	7 (21.9)	2 (18.2)	1.000
Cerebrovascular disease	5 (15.6)	6 (54.5)	**0.018** ^**b**^
Malignant tumor	6 (18.8)	0 (0)	0.312
Invasive procedures			
Tracheal intubation	20 (62.5)	3 (27.3)	**0.043** ^**b**^
Urinary catheter	22 (68.8)	4 (36.4)	0.080
Central venous catheter	17 (53.1)	3 (27.3)	0.138
Gastric tube	17 (53.1)	1 (9.1)	**0.014** ^**b**^
Prior antibiotic exposure			
Cephalosporins	3 (9.4)	2 (18.2)	0.589
β-lactamase inhibitors	17 (53.1)	4 (36.4)	0.337
Carbapenems	11 (34.4)	2 (18.2)	0.456
Fluoroquinolones	3 (9.4)	1 (9.1)	1.000
Clinical outcome			
30-day mortality^c^	11 (34.4)	2 (18.2)	0.456

Data are presented as frequencies and percentages, unless otherwise stated.

a Age is presented as median and interquartile range.

b Bold indicates *p*-value < 0.05.

c Death or treatment was discontinued due to poor prognosis.

**Figure 2:**
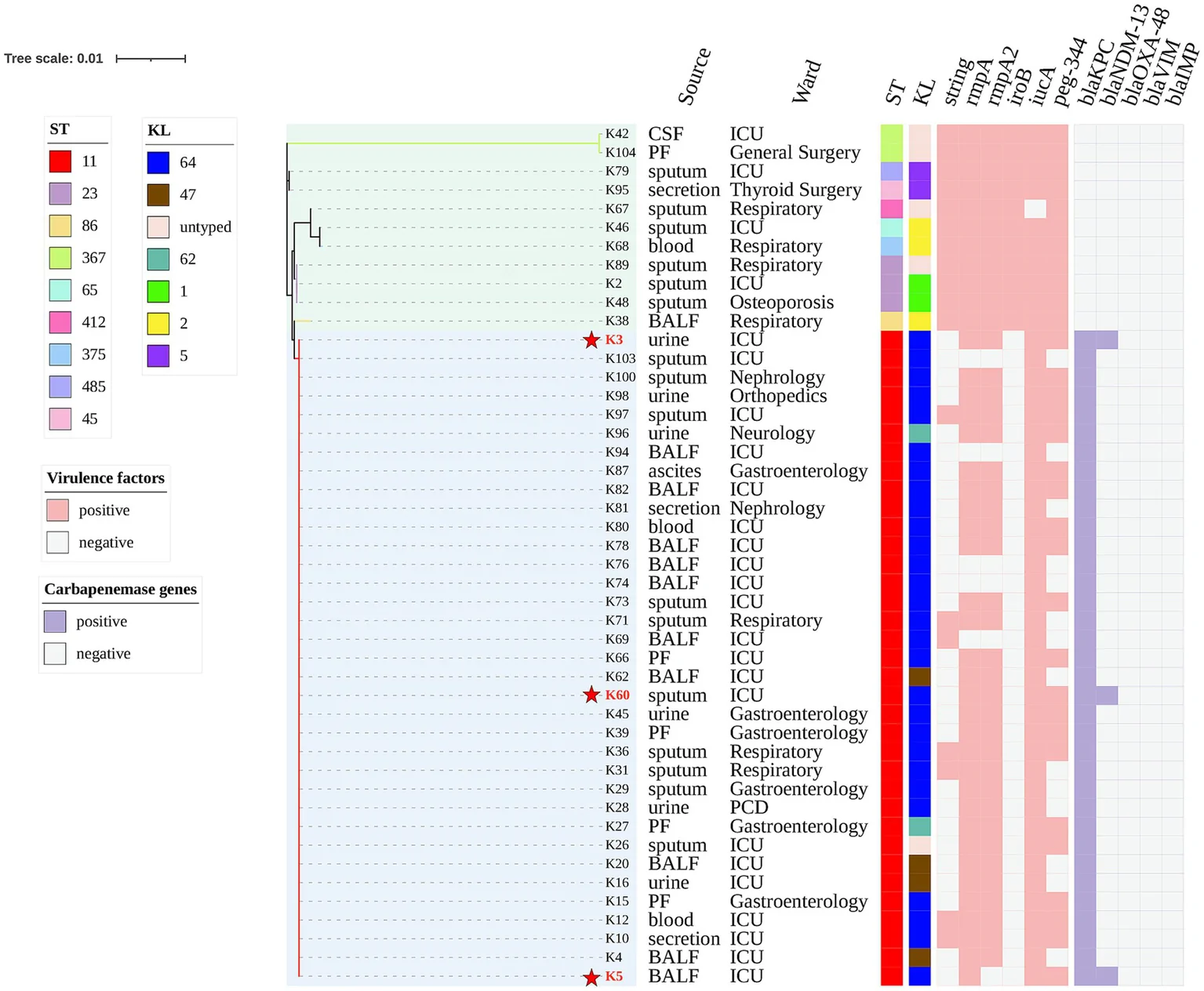
Phylogenetic tree based on concatenated sequences of the 7 MLST housekeeping genes from 46 genotype-defined hvKP strains. Trees are color-coded as follows: Light blue for genotype-defined hv-CRKP strains and light green for genotype-defined CS-hvKP strains. The red asterisk represents ST11-KL64 genotype-defined hv-CRKP strains co-harboring *bla*_KPC-2_ and *bla*_NDM-13_ genes. BALF, Bronchoalveolar lavage fluid; PF, Puncture fluid; CSF, Cerebrospinal fluid; PCD, Palliative care department.

The 46 genotype-defined hvKP strains were further divided into 35 genotype-defined hv-CRKP strains and 11 genotype-defined CS-hvKP strains (Figure 2). Most isolates were recovered from respiratory specimens (58.7%, 27/46). Molecular typing showed that genotype-defined hv-CRKP isolates were overwhelmingly dominated by the epidemic ST11-KL64 lineage. Notably, within this background, three ST11-KL64 genotype-defined hv-CRKP isolates were identified that co-harbored *bla*_KPC-2_ and *bla*_NDM-13_, which was phenotypically corroborated by a carbapenemase inhibitor enhancement test confirming concurrent production of KPC and NDM carbapenemases. Importantly, all three isolates were negative by the string test, indicating that hypermucoviscosity was not observed in these isolates.

### Clinical and molecular characteristics of three ST11-KL64 genotype-defined hv-CRKP isolates co-harboring *bla*_KPC-2_ and *bla*_NDM-13_

Three ST11-KL64 genotype-defined hv-CRKP isolates co-harboring *bla*_KPC-2_ and *bla*_NDM-13_ were recovered from three different ICU patients. These patients exhibited broadly comparable clinical characteristics, including critical illness, underlying comorbidities, and exposure to invasive procedures (Table 2). Regarding antimicrobial resistance, these three strains exhibited nearly identical resistance profiles. They were resistant to most conventional antimicrobial agents and susceptible only to key salvage agents, including tigecycline and aztreonam-avibactam (Supplementary Table S4). At the molecular level, the three isolates shared the same ST11-KL64 background and *bla*_KPC-2_/*bla*_NDM-13_ genotype, together with similar virulence gene profiles. Pairwise cgSNP distance analysis showed that K3, K5, and K60 differed by only 2–3 cgSNPs (Supplementary Table S5). These results indicate that the three ST11-KL64 genotype-defined hv-CRKP co-carrying *bla*_KPC-2_/*bla*_NDM-13_ belonged to a highly related local genomic cluster. K3 was therefore selected as the representative isolate for complete genomic and functional characterization.

**Table 2:** Clinical characteristics of 3 patients infected with ST11-KL64 genotype-defined hv-CRKP strains co-carrying *bla*_KPC-2_ and *bla*_NDM-13_.

Isolates	Age (Years)	Gender	Ward	Infection sites	Underlying diseases	Invasive procedures	Treatment	Clinical outcomes
K3^a^	74	Female	ICU	Urinary tract	Cerebral hemorrhage, central respiratory failure	Mechanical ventilation, urinary catheter, central venous catheterization, gastric tube	LVX, TGC, POL	Withdrawal of treatment
K5	20	Female	ICU	Lung	Botulism, acute respiratory failure	Mechanical ventilation, urinary catheter, central venous catheterization gastric tube	SCF, IPM	Improvement
K60	48	Female	ICU	Lung	Severe botulism, hypertension	Mechanical ventilation, urinary catheter, central venous catheterization, gastric tube	SCF, CIP, MIN, TGC	Withdrawal of treatment

LVX, levofloxacin; TGC, tigecycline; POL, polymyxin B; SCF, cefoperazone/sulbactam; IPM, imipenem; CIP, ciprofloxacin; MIN, minocycline.

a K3 was clinically diagnosed as a urinary tract infection rather than catheter-associated bacteriuria or colonization.

### Genomic characteristics of the representative strain K3

WGS revealed that strain K3 consisted of a single circular chromosome of approximately 5.5 Mb and six plasmids (Table 3). The virulence plasmid pK3-1-VIR carried main hypervirulence-associated genes. Most acquired multidrug resistance determinants were plasmid-borne, notably *bla*_KPC-2_ on pK3-2-KPC and *bla*_NDM-13_ on pK3-3-NDM, whereas 114 putative virulence-associated genes were identified on the chromosome (Supplementary Table S6). These genomic features indicate that K3 harbored distinct virulence and resistance plasmids, which were further characterized in subsequent comparative analyses.

**Table 3:** Genome information of the representative strain K3.

Name	Genome length (bp)	GC (%)	Coding region (bp)	Coding genes	Plasmid replicon type	Resistance genes	Virulence genes^a^	Accession number
Chromosome	5,489,319	57.38	4,764,522	5,229	/	*bla*_SHV_, *aadA2b*, *fosA6*, *sul1*	/	CP196480.1
pK3-1-VIR	224,483	50.07	185,736	262	IncHI1B(pNDM-MAR), repB	/	*rmpA*, *rmpA2*, *peg-344*, *iucABCD-iutA*	CP196481.1
pK3-2-KPC	127,428	53.07	103,224	187	IncFII(pHN7A8), IncR	*bla*_KPC-2_, *bla*_CTX-M-65_, *bla*_TEM-1_, *rmtB1*	/	CP196482.1
pK3-3-NDM	88,157	50.36	73,530	108	IncI1-I(Alpha)	*bla*_NDM-13_	/	CP196483.1
pK3-4	84,894	54.12	72,924	101	IncFII(pSDP9R)	*dfrA14*, *sul2*, *tet(A)*, *bla*_LAP-2_, *qnrS1*	/	CP196484.1
pK3-5	11,970	55.59	8,571	13	ColRNAI	/	/	CP196485.1
pK3-6	5,596	51.13	3,876	10	ColRNAI	/	/	CP196486.1

a Only virulence genes located on plasmids are included in this table.

### Analysis of the virulence plasmid from strain K3

**Figure 3:**
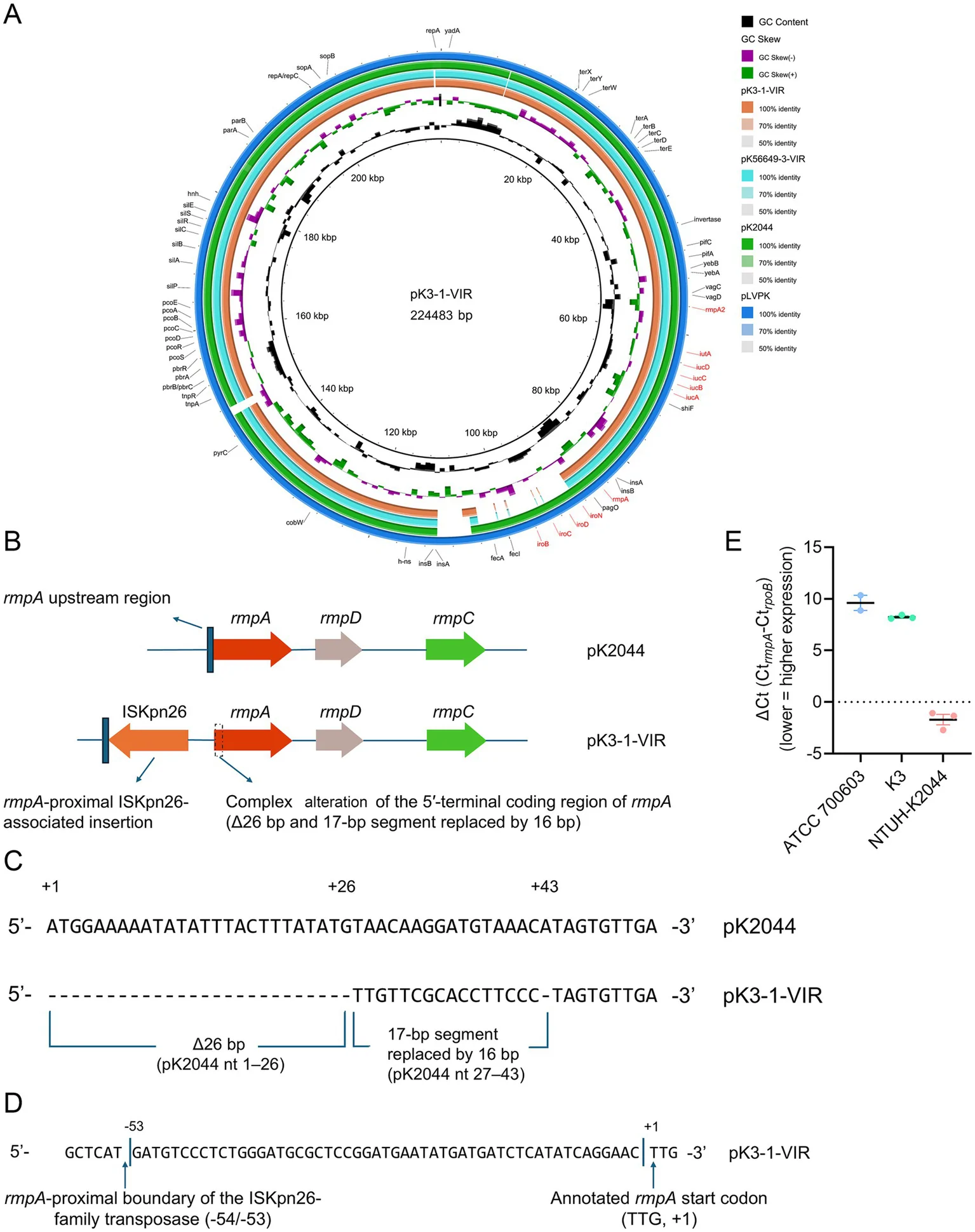
Comparative analysis of the virulence plasmid pK3-1-VIR and structural/transcriptional abnormalities of *rmpA* in strain K3. **(A)** BRIG comparison of the virulence plasmid pK3-1-VIR with three representative plasmids, including pK2044 (NC_006625.1) and pLVPK (AY378100.1), as well as pK56649-3-VIR (CP168403.1). pLVPK was used as the reference virulence plasmid, and virulence-associated genes are highlighted in red. **(B)** Schematic comparison of the *rmpADC* locus between pK2044 and pK3-1-VIR. pK3-1-VIR exhibited an *ISKpn26*-associated insertion in the upstream region and alteration of *rmpA*. The dashed box indicates the altered 5′-terminal coding region of *rmpA*. **(C)** Nucleotide alignment of the 5′ coding region of *rmpA* between pK2044 and pK3-1-VIR. Nucleotide positions were numbered relative to the canonical *rmpA* sequence of pK2044, with the adenine of the ATG start codon designated as +1. **(D)** Nucleotide alignment of the *rmpA*-proximal boundary of the *ISKpn26*-family transposase coding sequence in pK3-1-VIR. The boundary was located between positions −54 and −53 relative to the computationally K3 annotated TTG start codon of the disrupted *rmpA* allele, with the first thymine designated as +1. **(E)** Descriptive analysis of *rmpA* transcription in strain K3. Since *rmpA* expression was extremely low or undetectable in K3 and ATCC 700603, *ΔCt* values combined with a dot plot were used to characterize the transcriptional level of *rmpA*. Lower *ΔCt* indicates higher transcript abundance.

The virulence plasmid pK3-1-VIR from strain K3 was a 224,483-bp pLVPK-like plasmid with a GC content of 50.07% and carried the IncHI1B (pNDM-MAR) and repB replicons. Relative to the reference virulence plasmid pLVPK, pK3-1-VIR exhibited 98.07% nucleotide identity over 71.94% query coverage (Figure 3A). Despite the absence of the *iroBCDN* salmochelin locus, pK3-1-VIR retained major virulence-associated genes, including *rmpA*, *rmpA2*, *iucABCD-iutA*, and *peg-344*. Notably, comparative analysis revealed an *rmpA*-proximal *ISKpn26*-associated insertion and a complex alteration of the 5′-terminal coding region of *rmpA* (Figure 3B). Relative to pK2044, nucleotides 1–26 were deleted, whereas the 17-bp segment at positions 27–43 was replaced by a 16-bp sequence (Figure 3C). An *ISKpn26*-family transposase was inserted upstream of the disrupted *rmpA* allele in pK3-1-VIR (Figure 3D). *rmpA* transcript abundance in K3 was substantially lower than that in NTUH-K2044 (Figure 3E).

### Analysis of the resistance plasmids carrying *bla*_KPC-2_ and *bla*_NDM-13_ from strain K3

**Figure 4:**
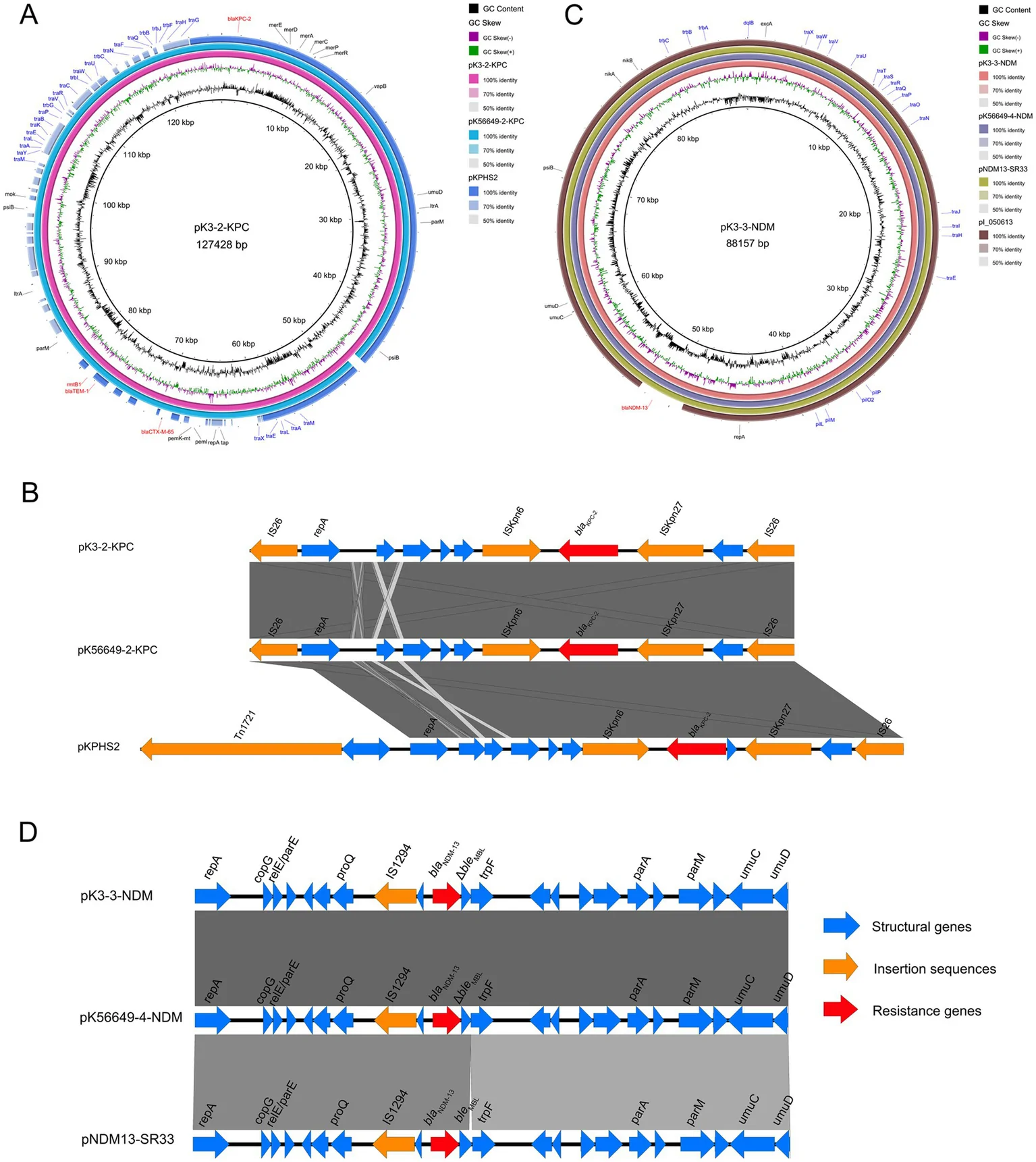
Comparative analysis of the resistance plasmids pK3-2-KPC and pK3-3-NDM in strain K3. **(A)** BRIG comparison of pK3-2-KPC with the *bla*_KPC-2_-bearing plasmids pKPHS2 (CP003224.1) and pK56649-2-KPC (CP168402.1), using pK3-2-KPC as the reference plasmid. Resistance genes are highlighted in red, and conjugation transfer-related genes are highlighted in blue. **(B)** Synteny analysis of pK3-2-KPC. Gray shading indicates homologous regions among the compared plasmids. **(C)** BRIG comparison of pK3-3-NDM with IncI1 plasmids, including pK56649-4-NDM (CP168404.1), pNDM13-SR33 (NZ_CP092912.1), and pI_050613 (CP019215.2), using pK3-3-NDM as the reference plasmid. **(D)** Synteny analysis of pK3-3-NDM.

The *bla*_KPC-2_-harboring plasmid pK3-2-KPC was a 127,428-bp plasmid with a GC content of 53.07%. Compared with pKPHS2 (CP003224.1), pK3-2-KPC carried an IncFII(pHN7A8)/IncR backbone rather than an IncFII(K)-type backbone and lacked a complete conjugative transfer region, including *traJ*. The plasmid also carried multiple resistance genes, including *bla*_KPC-2_, *bla*_CTX-M-65_, *bla*_TEM-1_, and *rmtB1* (Figure 4A). Relative to pK56649-2-KPC (CP168402.1), pK3-2-KPC exhibited 94.70% nucleotide identity and 100% query coverage. The genetic environment of *bla*_KPC-2_ was mainly organized as *IS26*-*ISKpn6*-*bla*_KPC-2_-*ISKpn27*-*IS26* (Figure 4B), showing high similarity to that of pK56649-2-KPC.

The *bla*_NDM-13_-harboring plasmid pK3-3-NDM was an 88,157-bp IncI1-I(Alpha) plasmid with a GC content of 50.36%. Its backbone was shared with plasmids identified in multiple *Enterobacterales*, including *E. coli*, *Salmonella* spp., and *K. pneumoniae*, represented by pI_050613 (CP019215.2), pNDM13-SR33 (NZ_CP092912.1), and pK56649-4-NDM (CP168404.1; Figure 4C). Comparative genomic analysis showed that pK3-3-NDM exhibited 99.38% nucleotide identity with 100% coverage to pK56649-4-NDM and 99.36% identity with 99.89% coverage to pNDM13-SR33. The genetic context surrounding *bla*_NDM-13_ was highly conserved, with a core structure of *IS1294*-*bla*_NDM-13_-Δ*ble*_MBL_-*trpF* (Figure 4D), consistent with that of the two reference *bla*_NDM-13_ plasmids. Further comparison showed that pK3-3-NDM also shared the homologous core resistance module *bla*_NDM_-Δ*ble*_MBL_-*trpF* with the *bla*_NDM-5_-carrying IncX3 plasmid pNDM_MGR194 (KF220657.1; Supplementary Figures S1A,B). In silico analysis using oriTfinder identified a predicted *oriT* region, relaxase gene, T4CP, and T4SS-associated gene cluster (Supplementary Table S7).

### Close relatedness between K3 and the Beijing isolate K56649

**Figure 5:**
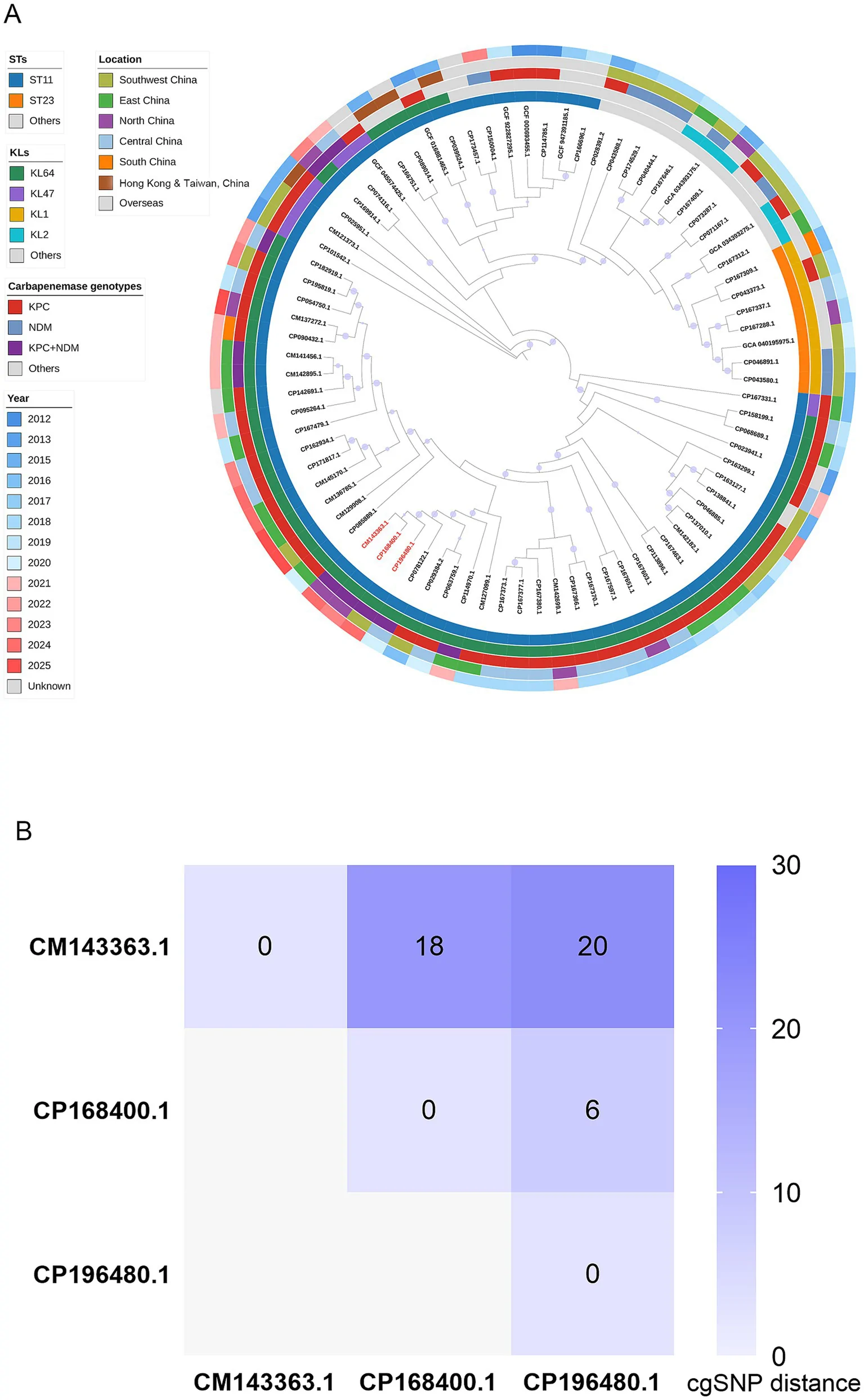
cgSNP phylogenetic analysis. **(A)** cgSNP-based phylogeny of 80 genomes, including three core comparator strains highlighted in red, together with 16 local background strains and an additional 61 domestic and international background strains. **(B)** Pairwise cgSNP distance matrix. CP196480.1 represents strain K3 from this study, while CP168400.1 and CM143363.1 represent the other two hv-CRKP strains in the core comparator set. Numbers indicate pairwise cgSNP distances, with smaller values indicating closer relatedness.

To explore the genetic relatedness of ST11-KL64 hv-CRKP strain K3 co-harboring *bla*_KPC-2_- and *bla*_NDM-13_-carrying plasmids, we reconstructed an expanded cgSNP phylogeny comprising 80 *K. pneumoniae* genomes. The cleaned full core-genome alignment had a length of 5,489,319 bp, of which 4,044,521 bp were callable core-genome sites across the 80 genomes. A total of 84,575 core polymorphic sites were identified before recombination filtering. The full pairwise SNP-distance matrix is provided in Supplementary Table S8. Strain K3 (CP196480.1) formed a strongly supported clade with the Beijing isolate K56649 (CP168400.1) and the Ningxia isolate A51998714 (CM143363.1; bootstrap = 100%; Figure 5A). Pairwise comparisons showed that K3 was most closely related to K56649, differing by only 6 cgSNPs, whereas K3 and A51998714 differed by 20 cgSNPs (Figure 5B).

### Plasmid transferability and stability

As shown in Table 4, conjugation using K3 as the donor and *E. coli* C600 as the recipient successfully generated the transconjugant K3T. The meropenem MIC of K3T was 32 μg/mL, representing a 16-fold decrease compared with that of K3. PCR confirmed that K3T carried only the *bla*_NDM-13_ gene, with a mean conjugation frequency of 5.213 × 10^−3^ transconjugants per recipient cell (range, 4.853 × 10^−3^ to 5.662 × 10^−3^). WGS analysis showed that K3T derived from an *E. coli* recipient had a genome size of approximately 4.62 Mb, with a GC content of 50.74%. Comparative genomic analysis showed that K3T (pNDM) exhibited 100% coverage to pK3-3-NDM (Supplementary Table S9), whereas no substantial coverage was detected for the pK3-2-KPC and pK3-1-VIR (Supplementary Figure S2). In contrast, *bla*_KPC-2_ was not detected in K3T under the tested conditions. After 10 days of serial passage in antibiotic-free medium, the retention rates of *bla*_KPC-2_ and *bla*_NDM-13_ in K3 were both 100% (30/30), and the retention rate of *bla*_NDM-13_ in K3T was also 100% (30/30).

**Table 4:** Transfer frequency and resistance profiles following conjugation of plasmid pK3.

Strains	Description	MIC of meropenem^a^	Carbapenemase	Carbapenemase genes	Conjugation frequencies^b^	
					Mean	Range
K3	Donor of the pK3	512	KPC, NDM	*bla*_KPC-2_, *bla*_NDM-13_	/	/
K3T	Transconjugant of pK3	32	NDM	*bla*_NDM-13_	5.213 × 10^−3^	4.853 × 10^−3^ ~ 5.662 × 10^−3^
C600	Recipient for conjugation	0.032	/	/	/	/

a The MIC unit is μg/mL. MIC, minimum inhibitory concentration.

b Conjugation frequency was calculated as the number of transconjugant CFUs divided by recipient CFUs.

### Attenuated virulence-associated phenotypes of strain K3

**Figure 6:**
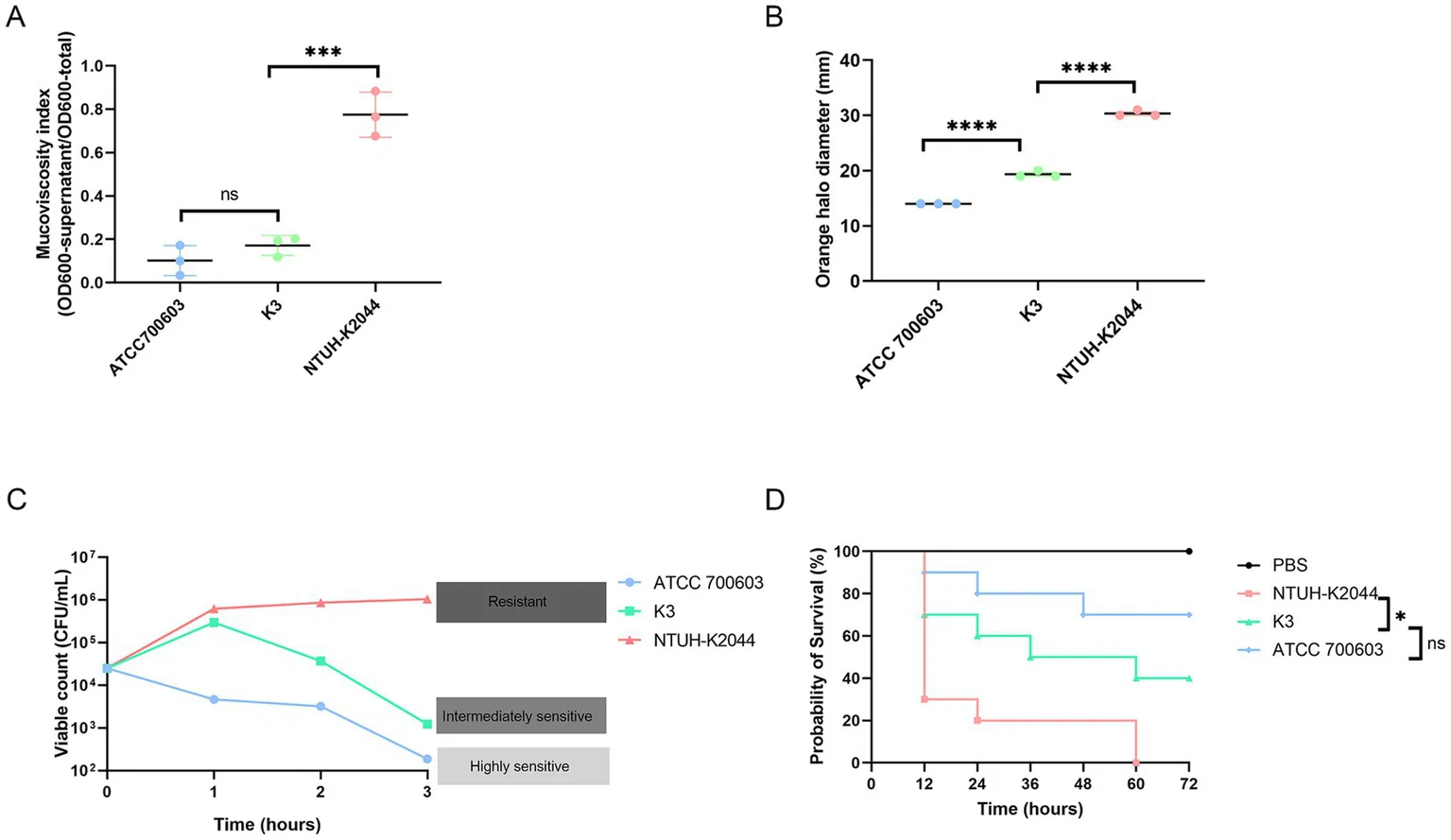
Virulence-associated phenotypes of strain K3. **(A)** Quantitative analysis of mucoviscosity. **(B)** Quantitative analysis of siderophore secretion. **(C)** Survival dynamics of different strains in the serum killing assay. **(D)** Survival curves in the *G. mellonella* infection model.

For the quantitative mucoviscosity assay, K3 exhibited significantly lower mucoviscosity than NTUH-K2044, with no statistically significant difference observed when compared to ATCC 700603 (mean 0.1717 vs. 0.7747, *p* < 0.001; mean 0.1717 vs. 0.1020, *p* = 0.54; Figure 6A). In the CAS assay, K3 showed intermediate siderophore production relative to NTUH-K2044 and ATCC 700603, with a mean halo diameter of 19.75 mm, compared with 30.25 mm for NTUH-K2044 (*p* < 0.0001) and 14.25 mm for ATCC 700603 (*p* < 0.001; Figure 6B). K3 also exhibited intermediate serum resistance (grade 4), whereas NTUH-K2044 was serum resistant (grade 6) and ATCC 700603 was serum sensitive (grade 2; Figure 6C). In the *G. mellonella* model, 40% of larvae infected with K3 survived at 72 h, indicating lower virulence than NTUH-K2044 but not significantly different from ATCC 700603 (Figure 6D).

## Discussion

Here, we identified three putative ST11-KL64 genotype-defined hv-CRKP isolates co-harboring *bla*_KPC-2_ and *bla*_NDM-13_. Pairwise cgSNP distance results showed that K3, K5, and K60 formed a highly related local genomic cluster. K3 was also closely related to K56649, consistent with recent shared ancestry. The *bla*_NDM-13_-carrying IncI1 plasmid was transferred by conjugation to C600 and *bla*_NDM-13_ showed stable retention under antibiotic-free serial passaging. Phenotypically, K3 displayed low mucoviscosity and attenuated virulence-associated phenotypes relative to classical hypervirulent NTUH-K2044. The low mucoviscosity in K3 was associated with structural alterations at the *rmpA* locus and reduced *rmpA* transcript abundance.

From a therapeutic perspective, co-production of KPC-2 and NDM-13 substantially limited the active *β*-lactam options. Current therapeutic guidance generally favors newer β-lactam/β-lactamase inhibitor regimens for KPC-producing *Enterobacterales*, whereas infections caused by NDM-producing isolates often require aztreonam combined with an avibactam-containing agent or cefiderocol, depending on susceptibility and drug availability (Tamma et al., 2024). K3, K5, and K60 exhibited nearly identical multidrug-resistant profiles, with resistance to common antibiotics, whereas aztreonam-avibactam and tigecycline retained *in vitro* activity, and polymyxin B MICs ranged from 0.5 to 2 μg/mL. The lack of ceftazidime-avibactam activity is consistent with the presence of NDM-13, which is not inhibited by avibactam. By contrast, aztreonam-avibactam exhibits activity against NDM-13. These findings highlight that antimicrobial susceptibility results should be integrated with carbapenemase genotypic or phenotypic characterization when selecting β-lactam-based therapy against hv-CRKP.

Beyond their similar antimicrobial susceptibility profiles, K3, K5, and K60 shared the ST11-KL64 background, co-harbored *bla*_KPC-2_ and *bla*_NDM_-13, and exhibited similar virulence-marker profiles, including *rmpA*, *iucA*, and *peg-344*. Pairwise cgSNP distance analysis revealed only 2–3 cgSNP differences among the three isolates. Collectively, these findings indicate that they constituted a closely related local genomic cluster consistent with recent shared ancestry. Nevertheless, the available epidemiological metadata were insufficient to reconstruct a direct transmission chain.

To our knowledge, reports of hv-CRKP carrying *bla*_NDM-13_ remain limited, including a Beijing outbreak cluster represented by K56649 (Zhang et al., 2024) and the Ningxia isolate A51998714 (Tao et al., 2025). In an expanded cgSNP phylogeny, K3, K56649, and A51998714 formed a strongly supported clade, with K3 differing from K56649 and A51998714 by 6 and 20 cgSNPs, respectively. Because fixed SNP thresholds are context dependent, the 6-cgSNP distance is best interpreted as evidence of close genomic relatedness consistent with recent shared ancestry, rather than as proof of a direct transmission event (Farzana et al., 2023; Hawken et al., 2022). Taken together, the low cgSNP distances support close genomic relatedness between K3 and K56649, whereas interregional transmission could not be established.

At the plasmid level, the principal functional finding was the experimentally demonstrated conjugative transfer of pK3-3-NDM. This 88,157-bp IncI1-I(Alpha) plasmid contained a predicted *oriT*, a putative relaxase, T4CP, and T4SS-associated gene cluster. In conjugation assays, pK3-3-NDM was transferred from K3 to C600 at a mean conjugation frequency of 5.213 × 10^−3^ transconjugants per recipient cell. The transconjugant K3T Illumina sequencing showed that 100% coverage of pK3-3-NDM, with no substantial coverage of pK3-2-KPC or pK3-1-VIR, supporting acquisition of pK3-3-NDM without detectable co-transfer of the *bla*_KPC-2_ or pLVPK-like plasmid. After approximately 100 generations without antimicrobial selection, *bla*_NDM-13_ was retained in all sampled colonies from both K3 and K3T. These findings provide direct evidence that pK3-3-NDM is conjugatively transferable and that the *bla*_NDM-13_ can be maintained under the tested conditions. Together with previous reports indicating that *bla*_NDM-13_ can be carried on transmissible IncX3 plasmids in *E. coli* and on IncI1 plasmids in *Salmonella* (Lv et al., 2016; Huang et al., 2022), our results indicate that *bla*_NDM-13_ has entered multiple plasmid backgrounds within *Enterobacterales*.

By contrast, transfer of *bla*_KPC-2_ was not detected in C600 conjugation system. pK3-2-KPC is an IncFII(pHN7A8)/IncR plasmid lacking a complete conjugative transfer region, with the key regulatory gene *traJ* notably absent. In F-like conjugative systems, TraJ serves as a major positive regulator of *tra* operon expression and is required for activation of genes involved in plasmid transfer (Lu et al., 2019). This structural feature therefore provides a plausible biological explanation for the inability of pK3-2-KPC to self-transfer under our experimental conditions. Nevertheless, non-self-transmissible resistance plasmids should not be regarded as completely non-mobile. Previous studies have shown that such plasmids can be mobilized by co-resident conjugative plasmids through trans-complementation of the missing transfer functions (O'Brien et al., 2015), and that IncFII/IncR backbones harboring *bla*_KPC_ may also serve as reservoirs for mobilizable resistance determinants. Separately, we observed that the IncI1 plasmid carrying *bla*_NDM-13_ and IncX3 plasmid carrying *bla*_NDM-5_ shared a conserved *bla*_NDM_-Δ*ble*_MBL_-*trpF* core resistance module. This local sequence conservation is compatible with possible modular sharing across distinct plasmid backbones. It should not be interpreted as evidence of cross-backbone mobilization because recombination junctions and direct movement of the segment were not demonstrated.

Virulence-associated phenotyping revealed discordance between carriage of hypervirulence-associated loci and the hypermucoviscosity phenotype. Although K3, K5, and K60 carried multiple virulence markers, including *rmpA*, *iucA*, and *peg-344*, all three were negative in the string test. Since the string test assesses hypermucoviscosity rather than hypervirulence, reliance on this assay alone may miss string-test-negative isolates carrying hypervirulence-associated loci (Russo et al., 2018; Russo et al., 2024). Quantitative analysis of K3 confirmed low mucoviscosity, which was significantly lower than that of NTUH-K2044 and not significantly different from that of ATCC 700603. Hybrid assembly identified a complex alteration at the 5′ end of *rmpA*, in which nucleotides 1–26 were deleted, and the 17-bp segment corresponding to positions 27–43 in pK2044 was replaced by a 16-bp sequence. In addition, the *rmpA*-proximal boundary of an *ISKpn26*-family transposase coding sequence was located 54 bp upstream of the computationally annotated TTG start codon. Previous studies have demonstrated that sequence variation within the upstream regulatory region of *rmpA* can alter promoter activity, *rmpA* expression, and mucoid phenotypes in ST11-KL64 backgrounds (Liu et al., 2023; Teng et al., 2024). However, the promoter boundaries of the K3 allele were not mapped, and the regulatory effect of this specific *ISKpn26*-associated alteration was not directly tested. To avoid interference from the altered 5′ region, the RT-qPCR amplicon was positioned downstream of the structural alteration, and K3 showed markedly lower downstream *rmpA* transcript abundance than NTUH-K2044. These findings suggest a possible relationship between alterations at the *rmpA* locus and the low-mucoviscosity phenotype of K3.

Beyond mucoviscosity, K3 exhibited attenuated virulence-associated phenotypes relative to NTUH-K2044. Its siderophore production and serum resistance were intermediate between NTUH-K2044 and ATCC 700603, whereas virulence in the *G. mellonella* model was lower than that of NTUH-K2044 but did not differ significantly from that of ATCC 700603. Similar genotype–phenotype discordance has been reported in *rmpA*/*rmpA2*-mutated isolates and in convergent isolates, many of which did not exhibit canonical hvKP-level virulence in murine models (Yu et al., 2015; Kochan et al., 2023). Nevertheless, these broader phenotypes should not be attributed solely to *rmpA*, because siderophore production, serum survival, and *in vivo* pathogenicity are multifactorial. Moreover, hypermucoviscosity and capsule abundance are related within the *rmpADC* regulatory system, in which *rmpD* is the proximal driver of hypermucoviscosity and *rmpC* regulates capsule expression (Walker et al., 2020; Lam et al., 2025). Therefore, our findings only support an association between *rmpA*-locus alterations and low mucoviscosity, but not a causal explanation for the broader attenuation of virulence.

Several limitations should also be acknowledged. Complete hybrid assembly and functional characterization were restricted to K3, precluding extrapolation of its plasmid architecture and *rmpA*-associated phenotype to K5 and K60. Although recombination-filtered cgSNP analyses supported close genomic relatedness among K3, K5, and K60 and between K3 and K56649, the absence of detailed temporal–spatial epidemiological data prevented reconstruction of direct local transmission chains or confirmation of interregional dissemination. Conjugation was assessed with a single *E. coli* recipient under one set of *in vitro* conditions. Therefore, non-detection of pK3-2-KPC transfer does not establish that this IncFII(pHN7A8)/IncR plasmid is intrinsically non-transferable or non-mobilizable. Plasmid size and resistance-gene localization were not independently corroborated by S1-PFGE and Southern blotting. Finally, without *rmpA* complementation or promoter-function assays, the association between *rmpA*-locus alterations and low mucoviscosity cannot establish causality or explain broader virulence attenuation.

## Conclusion

This study identified a highly related local ST11-KL64 cluster co-harboring *bla*_KPC-2_ and *bla*_NDM-13_, and K3 was capable of mediating conjugative transfer of the *bla*_NDM-13_-bearing IncI1 plasmid pK3-3-NDM. In K3, alterations at the *rmpA* locus and reduced *rmpA* transcript abundance were associated with low mucoviscosity, without establishing a causal contribution to broader virulence attenuation.

## Data Availability

Assemblies of strains K3, K5, K60 and K3T as well as raw sequencing reads of K3 in this study have been deposited in GenBank under BioProject PRJNA1291976. Other datasets generated for this study are included in the manuscript and Supplementary materials.

## Footnotes

1
https://cge.food.dtu.dk/services/PlasmidFinder/

2
https://www.mgc.ac.cn/VFs/main.htm

3
https://genepi.food.dtu.dk/resfinder

4
https://github.com/tseemann/snippy

5
https://github.com/tseemann/snp-dists
