# Supplementary material for: Genomic and functional characterization of ST11-KL64 hypervirulence-associated carbapenem-resistant Klebsiella pneumoniae co-harboring blaKPC-2 and blaNDM-13
Source: Front Microbiol. 2026 Aug 25;17:1874278. doi: 10.3389/fmicb.2026.1874278 (PMC13550197; doi:10.3389/fmicb.2026.1874278)
Supplement: Supplementary file 1 [file Data_sheet_1.docx]

**Table S1** Amplification primers used in this study.

| **Genes** | **Primer sequence (5′-3′)** | **Annealing temperature(◦C)** | **Size (bp)** |
| --- | --- | --- | --- |
| **Virulence genes** |  |  |  |
| *rmpA* | F: ATGTGGCTTGACGTTTCGGGGG | 55 | 160 |
|  | R: GCCGTGGATAATGGTTTACAATTCGGC |  |  |
| *rmpA2* | F: GGATGTGGCTTGACATTTCGGGGG | 55 | 227 |
|  | R: TTCATGGATGCCCTCCCTCCTG |  |  |
| *iroB* | F: ATCTCATCATCTACCCTCCGCTC | 58 | 235 |
|  | R: GGTTCGCCGTCGTTTTCAA |  |  |
| *iucA* | F: AATCAATGGCTATTCCCGCTG | 56 | 239 |
|  | R: CGCTTCACTTCTTTCACTGACAGG |  |  |
| *peg-344* | F: AAAGGACAGAAAGCCAGTG | 53 | 411 |
|  | R: CAATGACGAGGGGGATAATC |  |  |
| **Carbapenemase genes** |  |  |  |
| *bla*_KPC_ | F: ATGTCACTGTATCGCCGTCT | 55 | 893 |
|  | R: TTTTCAGAGCCTTACTGCCC |  |  |
| *bla*_NDM_ | F: ATGGAATTGCCCAATATTATGC | 55 | 813 |
|  | R: TCAGCGCAGCTTGTCGG |  |  |
| *bla*_OXA-48_ | F: GCGTGGTTAAGGATGAACAC | 56 | 438 |
|  | R: CATCAAGTTCAACCCAACCG |  |  |
| *bla*_VIM_ | F: GGTCGCATATCGCAACGCAGT | 55 | 636 |
|  | R: CGGCGACTGAGCGATTTTTG |  |  |
| *bla*_IMP_ | F: ATGAGCAAGTTATCTGTATTCTTTAT | 55 | 741 |
|  | R: TTAGTTGCTTAGTTTTGATGGTTT |  |  |
| **Housekeeping genes** |  |  |  |
| *rpoB* | F: GGCGAAATGGCWGAGAACCA | 50 | 1075 |
|  | R: GAGTCTTCGAAGTTGTAACC |  |  |
| *gapA* | F: TGAAATATGACTCCACTCACGG | 60 | 662 |
|  | R: CTTCAGAAGCGGCTTTGATGGCTT |  |  |
| *mdh* | F: CCCAACTCGCTTCAGGTTCAG | 50 | 756 |
|  | R: CCGTTTTTCCCCAGCAGCAG |  |  |
| *pgi* | F: GAGAAAAACCTGCCTGTACTGCTGGC | 50 | 718 |
|  | R: CGCGCCACGCTTTATAGCGGTTAAT |  |  |
| *phoE* | F: ACCTACCGCAACACCGACTTCTTCGG | 50 | 602 |
|  | R: TGATCAGAACTGGTAGGTGAT |  |  |
| *infB* | F: CTCGCTGCTGGACTATATTCG | 50 | 462 |
|  | R: CGCTTTCAGCTCAAGAACTTC |  |  |
| *tonB* | F: CTTTATACCTCGGTACATCAGGTT | 45 | 539 |
|  | R: ATTCGCCGGCTGRGCRGAGAG |  |  |
| **Capsular serotype** |  |  |  |
| *wzi* | F: GTGCCGCGAGCGCTTTCTATCTTGGTATTCC | 55 | 581 |
|  | R: GAGAGCCACTGGTTCCAGAACTTCACCGC |  |  |

**Table S3** qPCR primers used in this study.

| **Genes** | **Primer sequence (5′-3′)** | **Annealing temperature(◦C)** | **Size (bp)** |
| --- | --- | --- | --- |
| *rmpA* | F: TTCAGGGAAATGGGGAGGGT | 58 | 80 |
|  | R: CCGAAACGTCAAGCCACATC |  |  |
| *rpoB* | F: AAATCACCCAAGGCGACGAT | 58 | 119 |
|  | R: ACCCTTGTTACCGTGACGAC |  |  |

**Table S4** Selected clinically relevant antimicrobial susceptibility results for K3, K5, K60, and K3T strains.

| **Antimicrobial agents ^a^** | K3 | | | K5 | | | K60 | | | K3T | | |
| --- | --- | --- | --- | --- | --- | --- | --- | --- | --- | --- | --- | --- |
|  | MIC^b^ | | interpretation | MIC | interpretation | | MIC | interpretation | | MIC | interpretation | |
| **ATM** | >=64 | R | | >=64 | | R | >=64 | | R | <=1 | | S |
| **CAZ** | >=64 | R | | >=64 | | R | >=64 | | R | >=64 | | R |
| **CZA** | >=16 | R | | >=16 | | R | >=16 | | R | >=16 | | R |
| **MEM^c^** | 512 | R | | >=16 | | R | >=16 | | R | 32 | | R |
| **MNO** | 8 | I | | 8 | | I | 8 | | I | 1 | | S |
| **CIP** | >=4 | R | | >=4 | | R | >=4 | | R | 0.25 | | S |
| **TGC** | 1 | S | | 0.5 | | S | 0.5 | | S | 0.064 | | S |
| **POL** | 0.5 | I | | 2 | | I | 1 | | I | 0.5 | | I |
| **AZA inhibition zone diameter (mm)^d^** | 26 | S | | 27 | | S | 26 | | S | 40 | | S |

^a^ Broth microdilution was used for TGC and POL, while Kirby–Bauer disk diffusion was adopted for aztreonam-avibactam and automated system was used for the rest antibiotics.

^b^ The MIC values were expressed as μg/mL.

^c^ For meropenem MIC of K3 and K3T, an extended concentration range up to 512 μg/mL was used.

^d^ AZA discs contained 30/20 μg of the antimicrobial agents.

ATM, Aztreonam; CAZ, Ceftazidime; CZA, Ceftazidime-Avibactam; MEM, Meropenem; MNO, Minocycline; CIP, Ciprofloxacin; TGC, Tigecycline; POL, Polymyxin B; AZA, Aztreonam-Avibactam

**Table S5** Pairwise cgSNP distances among K3, K5 and K60 isolates.

| **Isolate** | **K3** | **K5** | **K60** |
| --- | --- | --- | --- |
| K3 | 0 | 3 | 2 |
| K5 | 3 | 0 | 3 |
| K60 | 2 | 3 | 0 |

**Table S6** Chromosome-associated virulence genes annotation for hv-CRKP strain K3.

| **VFs category** | **Virulence factors** | **Related genes** | **Chromosome (prediction)** |
| --- | --- | --- | --- |
| Adherence | Type I fimbriae | *fimK* | chr00894 |
|  |  | *fimH* | chr00895 |
|  |  | *fimG* | chr00896 |
|  |  | *fimF* | chr00897 |
|  |  | *fimD* | chr00898 |
|  |  | *fimC* | chr00899 |
|  |  | *fimI* | chr00900 |
|  |  | *fimA* | chr00901 |
|  |  | *fimE* | chr00903 |
|  |  | *fimB* | chr00904 |
|  | ECP | *yagW*/*ecpD* | chr04206 |
|  |  | *yagX*/*ecpC* | chr04207 |
|  |  | *yagY*/*ecpB* | chr04208 |
|  |  | *yagZ*/*ecpA* | chr04209 |
|  |  | *ykgK*/*ecpR* | chr04210 |
| Biofilm | Type 3 fimbriae | *mrkA* | chr00910 |
|  |  | *mrkB* | chr00911 |
|  |  | *mrkC* | chr00912 |
|  |  | *mrkD* | chr00913 |
|  |  | *mrkF* | chr00914 |
|  |  | *mrkJ* | chr00915 |
|  |  | *mrkI* | chr00916 |
|  |  | *mrkH* | chr00917 |
| Efflux pump | AcrAB | *acrA* | chr04052 |
|  |  | *acrB* | chr04053 |
| Regulation | RpoS | *rpoS* | chr01073 |
|  | RcsAB | *rcsB* | chr01586 |
|  |  | *rcsA* | chr01848 |
|  | PhoPQ | *phoP* | chr03319 |
|  | Fur | *fur* | chr03770 |
| Immune modulation | Capsule | / | chr01715, chr01716, chr01719, chr01721, chr01734, chr01737, chr01738, chr01739, chr01740, chr01741 |
|  | LPS | / | chr01735, chr01736, chr01743, chr01744, chr01745, chr01746 |
| Nutritional/Metabolic factor | Yersiniabactin | *fyuA*/*psn* | chr01812 |
|  |  | *ybtE* | chr01813 |
|  |  | *ybtT* | chr01814 |
|  |  | *ybtU* | chr01815 |
|  |  | *irp1* | chr01816 |
|  |  | *irp2* | chr01817 |
|  |  | *ybtA* | chr01818 |
|  |  | *ybtP* | chr01819 |
|  |  | *ybtQ* | chr01820 |
|  |  | *ybtX* | chr01821 |
|  |  | *ybtS* | chr01822 |
|  | Salmochelin | *iroE* | chr02605 |
|  |  | *iroN* | chr03089 |
|  | Aerobactin | *iutA* | chr03339 |
|  | Enterobactin | *entA* | chr03857 |
|  |  | *entB* | chr03858 |
|  |  | *entE* | chr03859 |
|  |  | *entC* | chr03860 |
|  |  | *fepB* | chr03861 |
|  |  | *entS* | chr03862 |
|  |  | *fepD* | chr03863 |
|  |  | *fepG* | chr03864 |
|  |  | *fepC* | chr03865 |
|  |  | *entF* | chr03866 |
|  |  | *fes* | chr03868 |
|  |  | *fepA* | chr03869 |
|  |  | *entD* | chr03870 |
| Secretion system | T6SS-I | *tssG* | chr02882 |
|  |  | *tssF* | chr02883 |
|  |  | *impA*/*tssA* | chr02884, chr02885 |
|  |  | *icmF*/*tssM* | chr02886 |
|  |  | / | chr02887 |
|  |  | / | chr02888 |
|  |  | *tle1* | chr02889 |
|  |  | *tli1* | chr02891, chr02892, chr02893, chr02894 |
|  |  | *vgrG*/*tssI* | chr02896 |
|  |  | *clpV*/*tssH* | chr02897 |
|  |  | *hcp*/*tssD* | chr02898 |
|  |  | *ompA* | chr02899 |
|  |  | *dotU*/*tssL* | chr02900 |
|  |  | *vasE*/*tssK* | chr02901 |
|  |  | *vipB*/*tssC* | chr02902 |
|  |  | *vipA*/*tssB* | chr02903 |
|  | T2SS | *gspS* | chr04340 |
|  |  | *gspB* | chr04341 |
|  |  | *gspD* | chr04344 |
|  |  | *gspE* | chr04345 |
|  |  | *gspF* | chr04346 |
|  |  | *gspG* | chr04347 |
|  |  | *gspH* | chr04348 |
|  |  | *gspI* | chr04349 |
|  |  | *gspJ* | chr04350 |
|  |  | *gspK* | chr04351 |
|  |  | *gspL* | chr04352 |
|  |  | / | chr04354 |
|  |  | / | chr04355 |
|  | T6SS-III | / | chr01995 |
|  |  | *impJ* | chr01996 |
|  |  | *dotU* | chr01997 |
|  |  | *ompA* | chr01998 |
|  |  | *vgrG* | chr02000 |
|  |  | *impG* | chr02018 |
|  |  | *impH* | chr02019 |
|  |  | *sciN* | chr02020 |
|  |  | *impF* | chr02021 |
|  |  | *impA* | chr02022 |
|  |  | / | chr02023 |
|  |  | / | chr02024 |

**Table S7** Predicted transfer-associated components of pK3-3-NDM identified by oriTfinder.

| **Component** | **Location** | **Strand** | **Locus tag/Gene name** | **Annotation** |
| --- | --- | --- | --- | --- |
| *oriT* region | 77,944–78,032 | - | / | Predicted origin of transfer |
| Auxiliary protein | 78,047–78,379 | + | XYI76394.1 | Transfer-associated auxiliary protein |
| Relaxase | 78,390–81,089 | + | ACSR4F_29915 | Putative relaxase |
| T4CP | 81,126–83,417 | - | ACSR4F_29920 | Putative type IV coupling protein |
| T4SS gene cluster | 1,677–34,668 | mixed | ACSR4F_29470–ACSR4F_29620 | Type IV secretion system-associated gene cluster |
| ARG | 50,877–51,689 | + | *bla*_NDM-13_ | Carbapenemase gene |

**Table S9** WGS-based comparative features between K3 and K3T strains.

| **Feature** | **K3 donor** | **K3T transconjugant** | **Interpretation** |
| --- | --- | --- | --- |
| Species background | *K. pneumoniae* | *E. coli* | Recipient background retained |
| *bla*_NDM-13_ | + | + | *bla*_NDM-13_ plasmid acquired |
| *bla*_KPC-2_ | + | - | *bla*_KPC-2_ plasmid not detected |
| *rmpA*/*rmpA2*/*iucA*/*peg-344* | + | - | Virulence plasmid not detected |
| pK3-3-NDM coverage | 100% | 100% | Consistent with acquisition |
| pK3-2-KPC coverage | 100% | Non-substantial coverage | No co-transfer evidence |
| pK3-1-VIR coverage | 100% | Non-substantial coverage | No co-transfer evidence |

**Figure S1** Comparative analysis of homologous *bla*_NDM_-associated regions in IncI1 and IncX3 plasmids. **A** BRIG alignment of IncI1 and IncX3 plasmids carrying *bla*_NDM_. The *bla*_NDM-13_ IncI1 plasmids include pK3-3-NDM (CP196483.1), pK56649-4-NDM (CP168404.1), pNDM13-SR33 (NZ_CP092912.1), and pI_050613 (CP019215.2), along with the *bla*_NDM-5_ IncX3 plasmid pNDM_MGR194 (KF220657.1). **B** Synteny analysis of the core modules between pK3-3-NDM and pNDM_MGR194.

**Figure S2** BRIG-based sequence coverage of the transconjugant K3T against the chromosome and major plasmids of donor strain K3. Circular comparisons were generated using the K3 chromosome or the indicated plasmid as the reference sequence. **A** K3 chromosome CP196480.1, **B** The *bla*_KPC_-2-bearing plasmid pK3-2-KPC (CP196482.1), **C** The *bla*_NDM-13_-bearing plasmid pK3-3-NDM (CP196483.1), **D** The virulence plasmid pK3-1-VIR (CP196481.1).
